# Phadiatop Seropositivity in Schizophrenia Patients and Controls: A Preliminary Study

**DOI:** 10.3934/publichealth.2014.2.43

**Published:** 2014-03-24

**Authors:** Olaoluwa Okusaga, Robert G. Hamilton, Adem Can, Ajirioghene Igbide, Ina Giegling, Annette M. Hartmann, Bettina Konte, Marion Friedl, Gloria M Reeves, Dan Rujescu, Teodor T. Postolache

**Affiliations:** 1 Mood and Anxiety Program, University of Maryland School of Medicine, Baltimore, MD USA; 2 Department of Psychiatry and Behavioral Sciences, The University of Texas Health Science Center at Houston, TX, USA; 3 Johns Hopkins Dermatology, Allergy and Clinical Immunology Reference Laboratory, Johns Hopkins University School of Medicine, Baltimore, MD USA; 4 St. Elizabeths Hospital, Psychiatry Residency Training Program, Washington, DC, USA; 5 Department of Psychiatry, University of Halle-Wittenberg, Germany; 6 Division of Child and Adolescent Psychiatry, University of Maryland, School of Medicine, Baltimore, Maryland, USA; 7 University of Maryland Child and Adolescent Mental Health Innovations Center, Baltimore, Maryland, USA

**Keywords:** schizophrenia, Phadiatop, atopy, allergen, allergy, IgE, immunology

## Abstract

There is a dearth of information on the association of atopy with schizophrenia. The few available studies used population-based registers to classify the atopy status of the patients but this strategy is not reliable. This study measured seropositivity with a multiallergen screen of allergen specific IgE antibodies in schizophrenia patients versus healthy controls. A subset of 66 schizophrenia patients and 34 healthy controls were randomly selected from a large comparative study of schizophrenia patients and controls. The Phadiatop multi-allergen screen was performed on sera from all the participants to assess their atopic status. Logistic regression was used to calculate the odds ratio for the association of schizophrenia with Phadiatop seropositivity as a measure of atopy. The prevalence of Phadiatop seropositivity was significantly lower (χ^2^ 4.59, *p* = 0.032) and there was a reduced odds ratio for atopy in schizophrenia patients relative to controls (OR 0.40; 95% CI 0.17 to 0.94, *p* = 0.036). Though limited by a relatively small sample size and potentially confounded by anti-psychotic medications, this study suggests that the prevalence of atopy is lower in patients with schizophrenia. Replicating these results in larger samples could add to our growing understanding of immunological implications in mental illness.

## 1. Introduction

Atopy is a genetically determined condition which predisposes an individual to a group of immunoglobulin E (IgE) antibody-mediated diseases including asthma, rhinitis and atopic dermatitis [1]. Increasing incidence and prevalence rates of atopic disorders, especially in western societies, have been hypothesized to relate to a shift in the immune response from a TH1 to a TH2 profile. This shift is postulated to be due to improved personal and environmental cleanliness, which has led to a reduced rate of exposure to fungal, microbial and infectious agents that typically mobilize TH1 inflammatory responses early in life; this is the previously called “hygiene hypothesis” [1],[2], now renamed the “old friends” hypothesis [3]. Previously, a TH2 hypothesis of schizophrenia has also been proposed by Schwarz et al [4], though in this instance, cytokine production was emphasized but no direct linkage to atopy was mentioned.

Schizophrenia is associated with a reduced life expectancy and some studies have reported that schizophrenia patients on average die 20 years earlier than the general population [5]. Comorbid medical conditions such as hypertension, diabetes, obesity, hypercholesterolemia and viral hepatitis are highly prevalent in schizophrenia and are risk factors implicated in the reduced life expectancy of schizophrenia [6]. A number of current treatment guidelines now emphasize the need for optimal monitoring of the physical health status of patients [7],[8]. In this vein, it is desirable to evaluate the association of atopy with schizophrenia since atopic disorders can impact significantly on an individual's physical health status. Evaluation of the association of atopy with schizophrenia is also important considering the fact that atopy or allergies have been associated with worsening of mental disorders, especially mood disorders [9]. For example, a spring peak of suicide has been consistently reported [10],[11] and one putative mechanism is the increased pollen count during spring which results in a worsening of allergic symptoms in sufferers potentially through production of mediators of inflammation that reach the brain and affect its ability to regulate behavior [12],[13]. Heightened allergic responses can potentially worsen mood symptoms [9] which could lead to suicide in vulnerable subjects. Animal studies have also shown that sensitization and exposure of rodents to aeroallergens results in behavioral changes including aggression and anxiety-like behavior with increased expression of genes for cytokines involved in allergy in the prefrontal cortex of the experimental animals [14],[15]. Similar elevations in the expression of TH2 cytokines have also been found in the orbitofrontal cortex of suicide victims [16].

The current literature on the association of atopic disorders with schizophrenia is relatively sparse and to our knowledge only three studies have been published to date. Weber et al [17] used data from the National Hospital Discharge Survey to evaluate the frequency of comorbid conditions in individuals with a primary diagnosis of schizophrenia and in those with a primary psychiatric disorder other than schizophrenia. The prevalence of asthma was greater in schizophrenia patients than those with another diagnosis. Chen et al [18] analyzed data from the Taiwan National Health Insurance Research Database for the years 2000 to 2002 and also found the prevalence rate of asthma to be greater in schizophrenia patients relative to healthy controls. The findings of Chen et al [18] and Weber et al [17] were recently replicated by Pedersen et al [19] who used a population-based cohort design and found an association between hospital contact for atopic disorders, especially asthma, and an increased risk of schizophrenia.

In terms of methods of confirmation of the atopy status of individuals, an expert group recently convened by the National Institute of Health has recommended that the Phadiatop multiallergen screen be used to define atopy [20]. The Phadiatop is a single serologic measurement of allergen specific IgE antibodies against the major aeroallergens and it is a test which characterizes an individual as atopic without specifying the precise allergen(s) to which a person is sensitized. It is important to note that all the previous studies that have evaluated the association between atopic disorders and schizophrenia have not given details on how the diagnosis of atopy was made in the study subjects, as they relied on data from population-based databases. The previous studies also relied on the population-based databases in classifying subjects as having a diagnosis of schizophrenia or not.

To specifically evaluate the association of atopy with schizophrenia and to overcome some of the limitations of previous studies, we have used the multi-allergen screen to detect serum IgE as a marker of the atopy status of all participants. We also used the Structured Clinical Interview for DSM-IV [21] to confirm the diagnosis of schizophrenia in patients. We aimed to compare prevalence rates of atopy between schizophrenia patients and healthy controls and hypothesized that atopy will be more prevalent among schizophrenia patients relative to healthy controls.

## 2. Materials and Method

### 2.1 Study Population

The participants in this study were a subsample of a larger study involving 950 schizophrenia patients and 1000 healthy controls [22]. From this master cohort, 66 schizophrenia patients and 34 healthy controls were randomly selected. The decision to select only 100 participants for the present study was based on the availability of funds for this preliminary add-on project. All the participants in the original study were Caucasian and residing in the Munich area of Germany. Patients were recruited from both inpatient and outpatient settings and the diagnosis of schizophrenia was confirmed using the Structured Clinical Interview for DSM-IV (SCID) [21]. Individuals diagnosed with schizoaffective disorder, schizophreniform disorder, substance-induced psychosis and psychotic disorder not otherwise specified were excluded. All the patients were treated with antipsychotic medication. The names and addresses of control participants were obtained from the Munich register and they were contacted by mail.

Written informed consent was obtained from all the participants after the study procedures were explained to them in detail. The local ethics committee of Ludwig Maximilians University, Munich, Germany approved the study and it was considered exempt by the Institutional Review Board of the University of Maryland School of Medicine Baltimore, Maryland, USA. The Phadiatop was performed on all sera using the ImmunoCAP250 autoanalyzer (Thermofisher Scientific/Phadia division, Kalamazoo, MI, USA).

### 2.2 Screening for allergic sensitization in the subjects

The Phadiatop is a single serological test that detects IgE antibody to a balanced mixture of 10 major aeroallergens on a single allergosorbent which covers the principal pollens, molds, dust mites, and pet epidermals, but not foods, drugs, or venoms [23],[24]. The Phadiatop is considered the single most accurate screening assay to delineate the atopic status of an individual. It does not, however, identify the precise allergen specificity to which the individual is sensitized (IgE antibody positive). Clinically, the presence of IgE antibody to any of the immobilized allergens is used to identify a positive atopic status. The reported IgE antibody level in kUa/L which represents a sum of the IgE antibody levels to all the immobilized allergens is not considered to have clinical value. A positive result indicates the presence of IgE antibody to one or several of the allergens specificities and the existence of an atopic state which is considered a risk factor for developing allergic disease. Despite the 0.1 kUa/L analytical sensitivity of the Phadiatop, we used a conservative cut point of 0.35 kUa/L to classify patients and controls as Phadiatop positive or negative.

### 2.3 Statistical analyses

Age and gender differences between patients and controls were evaluated using t-test and chi square analysis. Logistic regression was used to calculate the odds ratio for the association of schizophrenia with Phadiatop seropositivity as a measure of atopy. The statistical significance level was set at < 0.05 and all tests were 2-tailed. We performed all the statistical analyses using IBM SPSS version 20 (Armonk, NY: IBM Corp).

## 3. Results

### 3.1 Sample characteristics

Patients had a mean age of 42.2 ± 12.8 years while healthy controls had a mean age of 41.5 ± 14.1 years. Age did not differ statistically between the groups (*p* = 0.808). No gender difference was noted between the patient and control groups, with 60.6 % and 61.8% of the patients and controls, respectively, being male (*p* value for difference = 0.91).

### 3.2 Phadiatop seropositivity status between groups

The seropositivity prevalence based on the Phadiatop measurement was significantly lower in the schizophrenia patients relative to controls (χ^2^ 4.59, *p* = 0.032).There was a reduced odds ratio of Phadiatop seropositivity in schizophrenia patients relative to controls (OR 0.40; 95% CI 0.17-0.94, *p* = 0.036).

**Table 1.** Demographic variables, geometric mean IgE and Phadiatop status of schizophrenia patients and controls.

Characteristics	Healthy Controls (*n* = 34)	SchizophreniaPatients (*n* = 66)	*p*-value*
Age, years (mean ± SD)	41.5 ± 14.1	42.2 ± 12.8	0.81
Gender male, n (%)	21 (61.8)	40 (60.6)	0.91
Geometric mean Phadiatop (± SD)	0.91 ± 2.25	0.76 ± 2.26	0.289
Phadiatop status (n, %)			
Positive	20 (58.8)	24 (36.4)	
Negative	14 (41.2)	42 (63.6)	0.032

* χ^2^ test for categorical variables, t-test for continuous variables

## 4. Discussion

To our knowledge, this is the first study to evaluate an association between atopy and schizophrenia using the Phadiatop multiallergen screen. Schizophrenia patients had reduced odds ratio of atopy as assessed by Phadiatop seropositivity. Our findings contrast with those reported in the three previously published studies of atopic disorders in schizophrenia [17]–[19].

The differences in our findings with those from previous studies could be due to methodological differences. For instance, all the previous studies [17]–[19] used only clinical history based information to define allergic (atopic) disorders. However, asthma can be extrinsic (allergic) and intrinsic (non-allergic) [1], and therefore the history which is based on clinical records alone is an unreliable measure of atopy. The presence of IgE antibody, as evaluated in the current study, is considered by an international panel [20] to be a more robust indicator of allergic sensitization and atopic status. We have also included only SCID-diagnosed schizophrenia patients and excluded individuals with other psychotic disorders. The relatively high specificity of definition of atopy and reduced heterogeneity of the schizophrenia group in the current study could have contributed to the results being different from the three previously published ones. Conversely, it is possible that since the Phadiatop detects IgE antibody to the major 10 allergen groups that are known to cover 80% of allergic sensitization, we missed some specificities. This, however, would be expected to change the atopic status prevalence only slightly.

It would be worthwhile to investigate the possibility that antipsychotic medications may have attenuated the allergic response in schizophrenia patients. Even though a shift to a TH2-like immune reactivity was proposed to be present in schizophrenia [4], a recent meta-analysis found less consistent evidence in favor of a TH2 hypothesis but more evidence for a TH1 response in schizophrenia [25]. Our finding of reduced prevalence of atopy in schizophrenia may therefore be reflective of a shift from a TH2 to a TH1 response, though this idea is speculative since we did not measure and compare markers of TH1 response (e.g. INF-γ, TNF-α, IL-12) between schizophrenia patients and controls.

In favor of the hypothesis that schizophrenia might be associated more with a TH1 response rather than a TH2 response is the observation that schizophrenia is more common among the offspring of women who had infections during pregnancy [26],[27]. Since early exposure to infection may potentially be protective against atopic manifestations [28], it maybe that the increased prevalence of maternal infections in the mothers of schizophrenia patients has resulted in a reduced prevalence of atopy in this population. Furthermore, there is an increased prevalence of Toxoplasma gondii antibodies in patients with schizophrenia compared to controls [29] and there is a negative association between Toxoplasma gondii exposure and allergic sensitization [30],[31].

The relatively small sample size, non-inclusion of unmedicated schizophrenia patients (which would have enable us rule out potential confounding by antipsychotic medication) and the cross sectional design are some of the limitations of this study. The inclusion of only Caucasian subjects of German descent also limits the generalizability of our study results. The strengths of this study include the use of an objective and accurate screening assay to delineate the atopic status of the subjects [20] and the use of the Structured Clinical Interview for DSM-IV (SCID) [21] to confirm the diagnosis of schizophrenia in patients.

In conclusion, we have presented data that suggest a lower prevalence of atopy in schizophrenia patients relative to healthy controls. Certain conditions with immune implications such as certain malignancies and rheumatoid arthritis have also been negatively associated with schizophrenia. It may be that specific immune abnormalities involved in schizophrenia may represent factors of resilience in these conditions, or, alternatively, that immune abnormalities that predispose patients to atopy, rheumatoid arthritis and certain malignancies may moderate inflammatory pathways involved in schizophrenia. Our results would need confirmation in larger longitudinal studies, and cross-sectional studies involving unmedicated patients. Future studies should also include questionnaire on symptoms as well as skin tests (if possible) on patients and controls. If confirmed this finding could broaden our understanding of immune abnormalities associated with and implicated in schizophrenia.
